# The health-related quality of life of Syrian refugee women in their reproductive age

**DOI:** 10.7717/peerj.9990

**Published:** 2020-09-23

**Authors:** Manar Nabolsi, Reema Safadi, Carolyn Sun, Muayyad Ahmad, Du’a Al-Maharma, Suhaila Halasa, Mohammad Saleh, Jennifer Dohrn

**Affiliations:** 1 School of Nursing, The University of Jordan, Amman, Jordan; 2 Hunter Bellevue School of Nursing, Hunter College, NY, United States of America; 3 School of Nursing, Columbia University, NY, United States of America

**Keywords:** HRQoL, Refugee, Women, Syrian, Reproductive health, Jordan

## Abstract

**Background:**

Health-Related Quality of Life (HRQoL) for refugee women in reproductive age is highly affected by physical, political, psychosocial and environmental conditions in countries of asylum. HRQoL is enormously affected by the satisfaction of this vulnerable group with the physical, psychological, emotional and social care services provided in this critical time. Therefore, this study aimed toassess the HRQoL among Syrian refugee women of reproductive age living outside camps in Jordan.

**Methods:**

A cross-sectional correlational study was conducted with a convenience sample of 523 Syrian refugee women in the host communities in Jordan.Health-related quality of life (HRQOL) was measured using the short-form 36 (SF-36) questionnaire.

**Results:**

Significant negative correlations were found between SF-36 individual subscales score and the length of marriage, the number of children, parity and family income. The strongest correlations were between pain scale and length of marriage (*r* =  − .21), and between Energy/Fatigue and ‘number of children’ (*r* =  − .21). Conversely, antenatal care was positively correlated with physical, role emotional, pain, and general health. Physical functioning and general health were predicted significantly with less years of marriage, younger age at marriage, less violence and by higher family income.

**Conclusion:**

This study suggests low HRQoL scores for women of reproductive age across all domains. Several factors such as years of marriage, age at marriage, the number of children, violence, antenatal care and family income affected the women’s general health. The provision of appropriate and accessible reproductive and maternal healthcare services in antenatal visits is critical for ensuring the immediate and long-term health and wellbeing of refugee women and their families.

## Introduction

The conflict in Syria started in 2011 and was declared by the United Nations as one of the world’s worst humanitarian crises in the twenty-first century and a public health disaster (Baker, 2014). Because of this crisis, an estimated 6.3 million Syrian refugees were forcibly removed from their homes to other countries. About 5.6 million of these refugees fled to neighboring countries, including Lebanon, Turkey, and Jordan (United Nations High Commissioner for Refugees, 2019).

Jordan hosts the second-highest number of refugees (87 per 1,000 inhabitants) and is the sixth-highest refugee-hosting country in the world. At the time of this study (2018), 83% of Syrian refugees in Jordan live in urban areas, and only a smaller percentage of refugees were living in refugee camps concentrated mainly in the north of Jordan (UNHCR, 2018b).The largest proportion of Syrian refugees are living in four major cities, Amman: (34.42%), Irbid (27.14%), Mafrak (16.43%) and Zarka, (13.85%) (Higher Population Council, 2016). With limited health care resources as a lower-middle income country, promoting and improving refugees’ health is taxing to the existing health care system of Jordan. In fact, while Jordan had previously been categorized as an upper-middle income country, in 2017 Jordan was reclassified as a lower-middle income country, in part because of a growing population including refugees (World Bank, 2017).

The UNHCR figures of (2019) reported that women accounted for 49.9% of total Syrian refugee men and women residing out of camps in Jordan. Of this population (49.9%, 23.9% were women between the ages of 18–59 years old. In a world where people are forcibly displaced because of conflict or persecution, women and children are most vulnerable. Refugee women of reproductive age bear a disproportionate share of suffering and hardship due to displacement, war, and conflict situations requiring additional attention to maintain their physical, social and psychological wellbeing. Health issues such as mental health, reproductive health, communicable and non-communicable diseases are a priority for health care services for this population (Doocy et al., 2015; Hollander et al., 2016; Khawaja et al., 2017; Nelson-Peterman et al., 2015).

Several health issues arise when women escape conflicts and seek refuge in another country. Refugee women of reproductive age often encounter challenges in receiving family planning and perinatal care (Mortazavi et al., 2014; Reese Masterson et al., 2014; Sadat et al., 2013). Lack of healthcare at this age may expose women to pregnancy and obstetric complications after birth. Several studies among Afghans, Somalis, Iraqis and Syrian refugees suggest that women face physical and mental health challenges (Alemi et al., 2016; Bogic, Njoku & Priebe, 2015; Gerritsen et al., 2006; Ghumman, McCord & Chang, 2016; Hassan et al., 2016; Lillee, Thambiran & Laugharne, 2015; Maximova & Krahn, 2010; Taylor et al., 2014). A study by Hassan et al. (2016) suggested that Syrian women experienced a wide range of mental health problems because of the war and immigration. In another study, Syrian refugee women had an increased risk of suffering from post-traumatic stress disorder (PTSD) when exposed to more than one trauma (Alpak et al., 2015). Alemi et al. (2016) demonstrated that migrants suffered depression, anxiety, and symptoms of psychological distress. In addition to mental and psychological health, in a study of Syrian refugees in Lebanon, Reese Masterson et al. (2014) found that Syrian women’s physical health was affected by several reproductive health problems such as menstrual irregularities, pelvic pain and infections.

Health-Related Quality of Life (HRQoL) is a broad concept covering physical, mental, emotional and social constructs that affect the HRQoL. It is based on a person’s level of satisfaction with their physical condition, emotional state, and family and social life, and takes into account functional aspects in a person’s life (Group, 1993; Wilson & Cleary, 1995). HRQoL is a major issue to be considered in a vulnerable group such as refugee women. Having extra stressors such as pregnancy and childbirth as an immigrant in a different physical, psychosocial and environmental condition puts them at higher risk for physical and mental problems. Additionally, situations like immigration, aggravated by pregnancy and other reproductive needs, low level of education, separation from spouses and children, lack of health insurance and unemployment create serious challenges to maintain adequate healthcare services that promote health and HRQoL in extraordinary conditions. Accordingly, the purpose of this study was to assess health related quality of life of Syrian refugee women in the reproductive age within six months of giving birth to an infant as they live outside the refugee camps in Jordan. This will assist health care providers in planning interventions that enhance health services and guide policy makers in implementing services to promote the health of this vulnerable group.

## Materials & Methods

### Design

A cross-sectional correlational survey was used in this study.

### Sample and setting

The population of this study comprises Syrian refugee women of reproductive age (14–49 years old) who had given birth within the last six months. A proportional quota sampling technique was used to select women who were living outside the refugee camps in Amman (the capital of Jordan), Irbid, Mafrak and Zarka. Selection was based on the percentage of Syrian refugee women in each city, the four major hosting cities of Syrian refugees (Higher Population Council, 2016).

A priori sample size calculation was conducted revealing a sample of at least 393 mothers based on a 0.05 two-tailed level of significance, an effect size = 0.20 (small), and a power = 0.80 using mean difference test (Cohen, 1992).

### Instruments

The questionnaire consisted of two parts. The first part included questions pertaining to Syrian refugee women sociodemographic characteristics such as age, marital status, educational level, religion, number of children, income level, health insurance, and employment status. The second part included a short survey of the Arabic version of SF-36 of the eight HRQoL dimensions. The SF-36 measures quality of life in eight health domains: physical functioning (10 items), role limitations due to physical health problems (4 items), role limitations due to personal or emotional problems (3 items), energy/fatigue (4 items), emotional well-being (5 items), social functioning (2 items), bodily pain (2 items), and general health perceptions (5items) (Ware Jr & Sherbourne, 1992).

SF-36 is a valid and reliable tool across diverse populations (McHorney et al., 1994). SF-36 Health Survey has been used among the general populations in health and illness conditions and among refugees such as Karenni, Afghani, Iranian and Somali refugees (Cardozo et al., 2004; Gerritsen et al., 2006).

The Arabic version form was used to measure the health status of the Arab population in Saudi Arabia, Tunisia, and Lebanon. The tool measurement showed high internal consistency (*α* 0.70–0.90) across the eight subscales (Sheikh et al., 2015). These results suggest that the SF-36 Arabic version of the questionnaire is a valid and reliable scale to measure the quality of life of the Arab population.

### Data collection

This report is part of a larger study that examined aspects of reproductive health practices of Syrian refugee women in Jordan. Twenty-seven research assistants (RAs) were recruited and trained by the research team on data collection that took place over a period of six months (January –July 2018). We recruited female RAs to ensure that women felt comfortable as they participated in this study. After training, the instrument and methods were tested on a sample of 30 Syrian refugee women and was found as clear and relevant. Data were collected in women’s homes using face-to-face interviewing techniques. Research assistants assisted illiterate participants by reading each question and completing the form together.

### Ethical considerations

Ethical approval was obtained from the University of Jordan and the Department of Statistics (DOS). The DOS’s approval was necessary to facilitate RAs access to the host community and collect data from participants. Participants were informed of the purpose and procedures of the study. They were assured of their rights to confidentiality and their right to voluntary participation and to decline participation without reprimands. They were informed that participation has no or minimal risk. Upon approval of participation, each woman was invited to sign a consent form in Arabic. An additional consent was sought from guardians of women under 18 years old. A copy of this form that included a contact number for the research team was given to participants in case they had additional questions.

### Data analyses

The statistical analysis was conducted using the Statistical Package for the Social Sciences (SPSS) version 23.0 for Windows (SPSS Inc. Chicago, IL, USA). The SF-36 survey scores were coded and scored using the guidelines provided by the RAND Corporation (i.e., transformed to positive score between 0 and 100 where the higher the score, the better the health). For example, for pain, a higher score indicates greater freedom from pain. Pearson correlation coefficient was used to examine the relationship between the total and subscale scores of the SF-36, and demographic variables. Multivariate linear regression analysis was used to identify predictors of Sf-36 subscales

## Results

### Characteristics of participants

A total of 523 Syrian refugee postpartum women participated in the study with a response rate of 98%. Age ranged from 16 to 44 years (M ± SD = 26.11 ± 6.11). Mothers’ age at marriage ranged from 12 to 37 years (M ± SD = 18.50 ± 3.71) with a duration of marriage ranging from 1 to 25 years (M ± SD = 7.6 ± 5.3). The number of children in the participants’ families ranged from 1 to 11 years (M ± SD = 3.21 ± 1.84). Family’s monthly income in Jordanian dinar (JD; 1 JD = 1.41 US dollars) ranged from 30 to 940 JD (M ± SD = 222.6 ± 105.45). The majority of the participants did not experience violence (93.5). Women who have experience abortion one or more times were 37.3% of the studied participants (Table 1).

**Table 1:** Characteristics of women and their living conditions (*N* = 523).

**Characteristics**	**Mean (SD)**	**n (%)**
**Age**	26.11 (6.11)	
**Years of marriage**	7.59 (5.26)	
**Age of women on marriage**	18.5 (3.71)	
**Family income**	222.6 (105.54)	
**Number of children**	3.2 (1.84)	
**Antenatal care**		
Yes		470 (89.9)
No		53 (10.1)
**Violence experience**		
Yes		34 (6.5)
No		489 (93.5)
**Abortion**		
Zero time		328 (62.7)
One time		113 (21.6)
Two times		51 (9.8)
Three times		22 (4.2)
Four and more		9 (1.8)
**Baby loss**		
Yes		50 (9.6)
No		473 (90.4)

Table 2 shows the mean, standard deviation, and the Cronbach’s alpha for the eight health dimension scales. Each of the subscales was scored between 0 and 100. The highest mean was for the physical functioning (66.18) while the lowest mean was for role limitations due to emotional problems (42.83). The reliability coefficients as demonstrated by Cronbach’s alpha for the eight health subscales ranged between .61 and .90.

Table 3 shows the bivariate correlations between the eight health scales with the examined sociodemographic variables. Most importantly, the eight scales showed a significant negative association with (1) years of marriage, (2) number of children, and (3) previous births. That is, the greater the number of years of marriage, number of children, and number of previous births have negatively affected women’s overall quality of life. Additionally, there were two demographic variables: (1) family income and (2) antenatal care that showed positive association with HRQoL scales. Family income was positively correlated with physical functioning (.12^∗∗^) and general health (.11^∗^). Antenatal care was positively correlated with physical (.09^∗)^, role emotional (11^∗)^, freedom of pain (.12^∗∗),^ and general health (.10^∗^), indicating that women who received antenatal care had better quality in the physical, role emotional, freedom of pain, and general health domains. That is, the higher the family income, and the increased number of antenatal visits, the better is the quality of life of women in the reproductive age. There was a negative correlation between violence and emotional wellbeing (*r* =  − .10). The strongest correlation between HRQoL scales and sociodemographic variables were between ‘pain scale’ and ‘years of marriage’ (*r* =  − .21), and between ‘Energy/Fatigue’ and ‘number of children’ (*r* =  − .21).

**Table 2:** Means and standard deviations for the health scales (*N* = 523).

Health scales	Means	Standard deviations		Cronbach’s Alpha
Physical functioning	66.18	28.50	.90	
Role physical	46.80	43.88	.90	
Role emotional	42.83	44.31	.88	
Energy - fatigue	46.24	20.78	.61	
Emotional wellbeing	53.76	20.47	.68	
Social functioning	62.86	27.71	.66	
Pain	56.12	30.17	.88	
General health	57.56	19.92	.61	

**Table 3:** Bivariate correlations between health scales and specific conditions for Syrian refugee women.

Health scales	Violence experience	Years married	Age married	Number of children	Family income	Previous birth	Previous abortion	Baby loss	Antenatal care
Physical functioning	−.02	−.18^**^	−.13^**^	−.15^**^	.12^**^	−.15^**^	−.05	.08	.09^*^
Role physical	.04	−.15^**^	−.06	−.13^**^	.05	−.12^**^	−.12^**^	.06	.04
Role emotional	.04	−.14^**^	−.08	−.11^*^	.07	−.10^*^	−.10^*^	.03	.11^*^
Energy-fatigue	.06	−.19^**^	−.06	−.21^**^	−.01	−.20^**^	−.04	.04	.073
Emotional wellbeing	−.10^*^	−.13^**^	−.09^*^	−.14^**^	.02	−.13^**^	−.09^*^	−.03	.06
Social functioning	.01	−.16^**^	−.03	−.12^**^	.04	−.12^**^	−.06	.01	.08
Pain	.03	−.21^**^	−.06	−.18^**^	−.01	−.17^**^	−.12^**^	.07	.12^**^
General health	−.10^*^	−.16^**^	−.08	−.15^**^	.11^*^	−.16^**^	−.13^**^	.03	.10^*^

**Notes.**

* *p* < 05.

** *p* < 01.

**Table 4:** Refugee women’s characteristics as predictors for the health conditions.

Predictors	Physical functioning	Role physical	Role emotional	Energy - fatigue	Emotional wellbeing	Social functioning	Pain	General health
	**Betat-statistics**	**Betat-statistics**	**Betat-statistics**	**Betat-statistics**	**Betat-statistics**	**Betat-statistics**	**Betat-statistics**	**Betat-statistics**
Years	−.20	−.12	−.17	−.04	−.03	−.20	−.18	−.08
married	−2.46^**^	−1.44	− − 1.94^*^	−0.49	−0.38	−2.43	−2.18^*^	−0.96
Age	−.13	−.06	−.08	−.07	−.09	−.04	−.07	−.07
married	−3.00^**^	−1.35	−1.71	−1.60	−1.96^*^	−0.85	−1.62	−1.66
Number of children	−.07	−.28	−.06	−.26	−.16	.06	−.19	.10
	−0.43	−1.56	−0.32	−1.50	−0.88	0.35	−1.09	0.58
Family	.11	.04	.06	−.02	.01	.04	−.02	.11
income	2.53^**^	0.97	1.24	−0.35	0.16	0.88	−0.38	2.48^**^
Previous birth	.09	.27	.10	.10	.06	−.01	.19	−.18
	0.54	1.57	0.60	0.59	0.32	−0.05	1.15	−1.04
Previous abortion	.01	−.09	−.07	−.01	−.07	−.02	−.08	−.08
	0.04	−1.99^*^	−1.50	−0.15	−1.52	−0.37	−1.83	−1.88
Baby loss	.05	.05	.01	.02	−.05	−.03	.04	−.01
	1.21	1.09	0.14	0.49	−1.08	−0.60	0.96	−0.07
Antenatal care	.05	.01	.08	.04	.03	.06	.09	.06
	1.21	0.26	1.79	0.86	0.71	1.24	2.08^*^	1.28
Violence experience	−.04	.03	.02	.04	.08	−.01	.01	−.09
	−0.91	0.69	0.55	0.92	1.84	−0.03	0.15	−2.12^*^
**R**	**.27**	**.21**	**.21**	**.23**	**.21**	**.18**	**.27**	**.27**
**R**^**2**^	**.07**	**.05**	**.04**	**.05**	**.04**	**.03**	**.07**	**.07**
**F**	4.54^**^	2.67^**^	2.60^**^	3.25^**^	2.60^**^	1.94^*^	4.10^**^	4.15^**^

**Notes.**

* *p* ≤ .05.

** *p* ≤ .01.

Refugee women’s characteristics as predictors for the health conditions are presented in Table 4. Before running the analyses, all the statistical assumptions for multiple linear regression were examined. None were violated. All the eight multiple regression models were significant in this study.

The selection of the nine predictors was based on its importance to the refugee women and the statistical correlation with the health scales. The highest explained variance was the same (*R*^2^ = .07) for physical functioning, pain, general health scales. Physical functioning was predicted significantly as better health with less years of marriage, younger age at marriage, and with higher family income. The variables (number of children, previous abortion, previous birth, and baby loss) showed poor prediction across all the health conditions. General health was predicted significantly with better family income and less violence. Freedom of pain was predicted significantly with less years of marriage and antenatal care.

## Discussion

This study explored the HRQoL of Syrian refugee women within six months of giving birth and who were living outside of the refugee camps in Jordan. The scores of HRQoL measurement scales and the subscales were compared with women in this study and women living in nearby Arab countries reported in other studies (Daher et al., 2011; Matalqah et al., 2018; Sabbah et al., 2003), taking into considerations that these studies may have had different methodologies and have been conducted in different socio-political circumstances. The results of the current study showed that Syrian refugee women living outside of refugee camps in Jordan scored lower on all domains compared to other women living in northern Jordan and Iraqi refugee women.

Additionally, Syrian refugee women scored lowest in general health scores compared to Jordanian and Iraqi refugees. This can be explained in light of the psychosocial and physical burden after having a new baby and not being well settled in Jordan, unlike Jordanian, Lebanese and Iraqi women.

The highest mean score of the SF-36 scale in the study was the physical functioning. This finding is similar to another study of the Arab populations including Jordanian and Lebanese people. In their study, Sabbah et al. (2003) and Doocy et al. (2015) showed that physical health scores were the highest, whereas, the emotional health scores were the lowest. In another study about the Iraqi refugees, women scored the highest in social functioning. It is probably that Iraqi women were better settled with time elapsing since migration to Jordan and have developed more social network that enabled them to cope with their needs compared to this study population (Daher et al., 2011).

In the current study, there was a decline in physical health with increasing years of marriage, number of children, and older age at marriage; and at the same time, factors that significantly predicted physical functioning were also less years of marriage, younger age at marriage, higher family income, and less violence, all of which are aligned with Mirghafourvand et al. (2016) study. Such findings may reflect women’s energy depleting with increasing requirements to fulfil their assumed role as a caretaker of the family members, a mother, and a wife with an increased number of children and responsibilities. An increasing age and family demands in unusual living conditions, such as in resettlement, may drain a woman’s energy and functioning levels.

The lowest SF36 score in this study was in role emotional. This result could point to the influence of their emotional condition and their inability to perform their roles as mothers and wives. Being a refugee in a different social and environmental context, compounded by having a new child, without social support, financial strains, and war-related stressful situations are aggravating factors that may likely contribute to women’s emotional distress. This finding is consistent with the findings of a study in Northern Jordan (Matalqah et al., 2018). Both populations (Jordanian and Syrian) are facing similar socioeconomic challenges in poverty and are surrounded by war and political turbulence in neighbouring countries. Alternatively, Iraqi refugee had their lowest score in energy and fatigue scales (Daher et al., 2011), which could be related to their prolonged refuge situation compared to Syrian refugees.

In this study, women who received antenatal care had better health (physical, role emotional, pain, general health). A significant positive correlation was found between income and antenatal visits with improved HRQoL subscale scores. Jordan is dedicated to providing reproductive health care services to refugees throughout the widespread mother and child care centres and mobile services (Saadallah & Baker, 2016). However, a study of Syrian refugees in camps reported that 23 percent of refugee women were unaware of these services, about 28 percent experienced unplanned pregnancies, and 17 percent did not access antenatal care during their pregnancy (Kohler, 2014).

The strongest correlation was between pain scale and length of marriage. In a study of Jordanian women Matalqah et al. (2018) found that Jordanian women suffered more pain and limitations due to pain compared with this study group, the Syrian refugee women. The other strong correlation was between energy/fatigue and number of children. Women in this study may be overwhelmed with their family’s needs, and in the case of refugee women with a large family and large number of children, lack of resources and support, and having a new child. It is no surprise that these responsibilities drain their energy and cause fatigue (Iwata et al., 2018; Schmied et al., 2017).This study suggested that participants’ energy and fatigue, and health in general were negatively affected by the increased number of children in the family; this may, in part, lead to increased financial burden. However, with increasing age and length of marriage, women usually develop better adaptation to the increased demands of the family. Age-related changes in coping and adaptation show a curvilinear pattern over the life course, with a peak in midlife and declines in older age (Robinson & Lachman, 2017).

The findings suggest a relationship between low socioeconomic status and poor health. Studies have shown that physical and mental health problems are highly prevalent in vulnerable populations, such as refugees and asylum seekers (Dorling, Mitchell & Pearce, 2007). It was also found that having health insurance has enhanced physical and emotional health. About two-thirds of the women (68.5%) in the current study did not have health insurance. As in 2018 and as a result of the national financial burden and the international funding cuts, the Jordanian Government and International organizations have reduced budget plans in providing health care services to refugees. Therefore, refugees have to pay about 80% of health care services, besides facing the challenges to access basic health care services (Doocy et al., 2015; Kohler, 2014). As a result, there will be an increasingly challenging situation to meet the needs of over 650,000 refugees in the country (UNHCR, 2018a).

Vulnerable women such as refugees in their postpartum period may undergo physical, emotional and social changes that may expose them to several health risks (Sadat et al., 2013; Tucker et al., 2010). In this critical period, women may need health care services that improve their HRQoL and wellbeing and prevent comorbidity and complications. The literature has shown that forced displacement of Syrians affected their HRQoL, including their physical (Doocy et al., 2015), psychological (Weinstein, Khabbaz & Legate, 2016), and social wellbeing (Sevinç et al., 2016). This study confirms those findings and highlights specifically the needs of women of reproductive age living outside refugee camps.

### Strengths and limitations

This is the first study to address the HRQoL of Syrian refugees’ women during a critical time, shortly after birth at a sensitive period when they needed more support, better health care services, while they were struggling with the refugee status.

A survey, self-report design is used in this study. In this design bias may affect the results as participants may be too embarrassed to reveal private details. Accordingly, we recommend further qualitative and longitudinal and study that can enhance our knowledge in examining the HRQoL of vulnerable women. Furthermore, the use of non-random sampling limited the generalizability of the findings. Further studies to include Palestinian refugee women is recommended.

## Conclusions

In this study, we found that the general health and HRQoL of Syrian refugee women were low compared to the findings of other studies of Arab women in the literature.

There is a relationship between low socioeconomic status and poor HRQoL. Several factors such as years of marriage, age at marriage, the number of children, violence, antenatal care and family income affected the women’s general health. The provision of optimal reproductive and maternal healthcare is critical for ensuring the health and wellbeing of refugee women and their families. For refugee women, access to maternity care influences their immediate and long-term health; it also impacts upon integration, attitudes to health and health-seeking behaviour, and may have health ramifications upon the next generations. Providing equitable access to quality reproductive health services for Syrian refugees and Jordanians poses enormous challenges for a small country like Jordan.

In conclusion, health services must be responsive to the health needs of refugee women in their reproductive age as they face several health challenges related to physical, mental and socioeconomic status. Nurses can play a major role in promoting the health of refugee women. Addressing barriers to seeking health care services and attending to health care needs and providing health education, affordable and accessible health care services can improve Syrian refugee women health and HRQoL. Further studies to include Palestinian refugee women is recommended.

##  Supplemental Information

10.7717/peerj.9990/supp-1 Supplemental Information 1 Raw Data

## Additional Information and Declarations

### Data Availability

The following information was supplied regarding data availability: The raw data are available as a Supplemental File.
